# Fabrication and Characterisation of Aligned Discontinuous Carbon Fibre Reinforced Thermoplastics as Feedstock Material for Fused Filament Fabrication

**DOI:** 10.3390/ma13204671

**Published:** 2020-10-20

**Authors:** Lourens Gerrit Blok, Marco Luigi Longana, Benjamin King Sutton Woods

**Affiliations:** Bristol Composites Institute (ACCIS), University of Bristol, Bristol BS8 1TR, UK; m.l.longana@bristol.ac.uk (M.L.L.); ben.k.s.woods@bristol.ac.uk (B.K.S.W.)

**Keywords:** short fibre, thermoplastic, composite, consolidation

## Abstract

In this work, aligned discontinuous fibre composite (ADFRC) tapes were developed and investigated as precursors for a novel 3D printing filament. ADFRCs have the potential to achieve mechanical performance comparable to continuous fibre reinforced composites, given sufficient fibre length and high level of alignment, and avoid many of the manufacturing difficulties associated with continuous fibres, e.g., wrinkling, bridging and corner radii constraints. Their potential use for fused filament fabrication (FFF) techniques was investigated here. An extensive down-selection process of thermoplastic matrices was performed, as matrix properties significantly impact both the processing and performance of the filament. This resulted in four candidate polymers (ABS, PLA, Nylon, PETG) which were used to manufacture ADFRC tapes with a *V*_f_ of 12.5% using the high performance discontinuous fibre (HiPerDiF) technology and an in-house developed continuous consolidation module. Tensile stiffness and strength up to 30 GPa and 400 MPa respectively were recorded, showing that a discontinuous fibre filament has the potential to compete with continuous fibre filaments.

## 1. Introduction

Carbon fibre reinforced plastics (CFRPs) are well known for their high specific mechanical properties and continue to attract considerable research interest. The manufacturing of these composite materials, however, is expensive as a high level of expertise is required on top of significant investments for equipment [1]. This slows down the growth of composite materials and therefore cheaper manufacturing methods are a key enabling technology for wider commercial uptake of composite and to enable shorter product development times [2,3].

Over the last few decades, rapid prototyping technologies have emerged using additive manufacturing to build up parts layer-by-layer (LbL) [2,4]. This allows direct fabrication of net-shape parts with new design freedom. Fused filament fabrication (FFF), also known as fused deposition modelling (FDM), is the most common LbL technique, where a part is build-up by the deposition of thermoplastic material through a nozzle, also known as 3D printing. A relatively new area of research is using fibre reinforced filament to create composite parts using a fully automated process [5,6,7].

Several review papers summarise the state-of-the-art on composite 3D printing [8,9,10]. An important parameter for 3D printing of fibre reinforced filament is the fibre length. Continuous fibre reinforced filaments show the highest performance but are limited in deposition freedom as the fibres are inextensible along the length [11]. Short fibre reinforced filaments are more versatile in terms of deposition freedom but lack mechanical performance as the fibres (~0.1 mm) are too short to reach their full strength [5,6,8].

The concept of critical fibre length must be introduced here to explain the mechanical performance of discontinuous or short fibre composites. For very short fibres in composites, the full fibre strength cannot be achieved as the fibre-matrix interface will fail before the fibre (fibre-pull out). The fibre length at which the failure mode changes from pull-out to fibre failure, i.e., the length that allows to exploit the full fibre strength, is defined as the critical fibre length [12,13,14].

**Figure 1:**
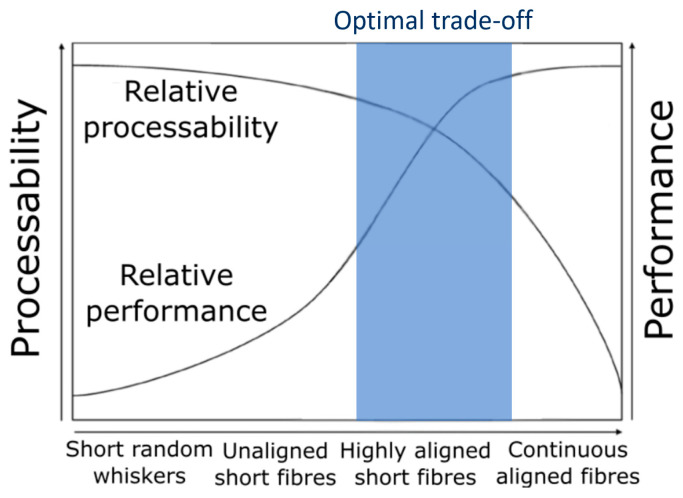
Balance between process ability and performance for different fibre architectures showing the optimal trade-off region, adapted from [18].

In this work, the use of thermoplastic aligned discontinuous fibre composites (ADFRCs) is proposed to improve the 3D printing of composites. ADFRC tapes are manufactured with fibres above the critical fibre length to be used as a 3D printing material feedstock. Previous work has shown that ADFRCs with fibres above the critical fibre length can reach a performance up to 85% of continuous fibre composites [15]. This should enable an optimum between processing and performance, as shown in Figure 1. Careful selection of the fibre length and matrix system allows for retention of most of the mechanical performance of a continuous fibre solution, without the need for cumbersome and restrictive fibre cutting and initial laydown procedures within the additive manufacturing process. This process may also be suitable for recycling of continuous fibre waste and enable a low-cost method for composite manufacture where rapid prototyping and a high degree of automation are required [16,17].

## 2. Materials and Methods

### 2.1. Matrix Selection Process

Depending on the application and manufacturing method, different thermoplastic polymers may be selected as the matrix of composite materials. Here, the focus is on extrusion-based additive manufacturing such as FFF. FFF is strongly influenced by rheological and thermal phenomena during printing which can be influenced in part by the printing setting, but which are ultimately governed by intrinsic material properties. During the FFF process, the material heats up, flows out of the nozzle and solidifies again after being extruded. Multiple studies have investigated heat transfer and extrusion problems in 3D printing [19,20,21,22,23].

**Figure 2:**
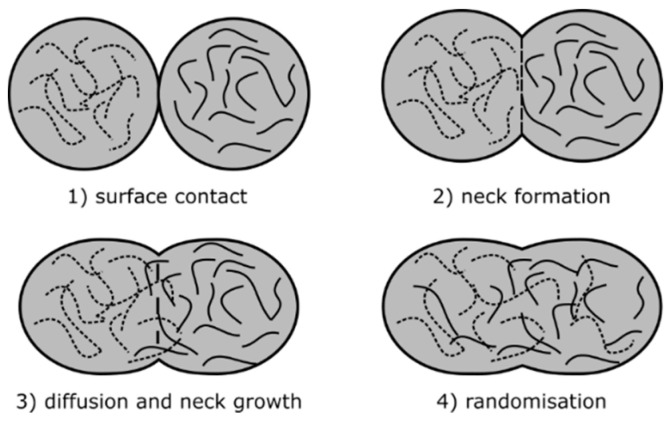
Schematic overview of the polymer sintering process, adapted from [23].

When the hot, viscous thermoplastic material is extruded it bonds with surrounding material through a mechanism known as polymer sintering. After contact, the newly deposited polymeric material coalesces with the previously deposited material in a process driven by surface tension and viscous flow for polymers above their glass and melt temperatures [24]. The amount of initial surface contact and the distribution of heat between two adjacent beads leads to the formation of a neck (Figure 2) as absorptive equilibrium is reached (a lower state of overall energy by minimizing surface area). This process is inhibited by the viscosity of the material. During neck formation, diffusion of the polymer chains occurs while the viscosity of the material increases as it cools down, slowing down the neck formation and diffusion process [23]. No external pressure is applied and the bond strength between printed tracks is determined by how well the polymer chains are entangled across the boundary [21,25].

Reptation theory, as introduced by de Gennes in 1971, describes the thermal motion of polymer chains as snake-like Brownian motion. This can be used to predict the reptation time, a characteristic time for the polymer chains to have completely moved to a new position [26,27]. The reptation time is an important characteristic time that relates to the level of (new) entanglement across an interface and can be used to predict the bond strength [25,27].

**Figure 3:**
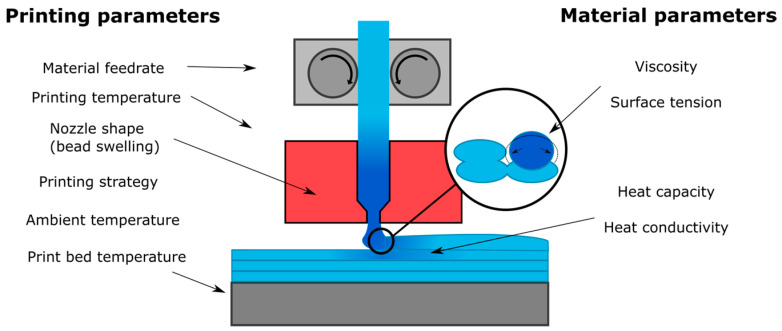
Main parameters for good surface contact and temperature conditions to enable optimal polymer sintering conditions.

The polymer sintering process is therefore sensitive to the viscosity (temperature dependent), thermal conductivity and heat capacity of the material, as well as the cooling rate (determined in part by the external environment). A higher temperature leads to better flow of the polymer melt and a higher mobility of the polymer chains, improving the polymer sintering and diffusion process. Similarly, a higher thermal conductivity would improve heat distribution, aiding the chemical bonding between filaments as previously deposited material heats up to improve the sintering process. At too high temperatures, however, the polymer may degrade, and dimensional accuracy may decrease because of the increased flow. Figure 3 summarises the printing and material parameters that are expected to influence the 3D print quality, mapped to the different stages of the printing process.

The addition of fibre reinforcement increase the viscosity of the bulk material in molten state [28] and they increase the heat capacity and heat conductivity [29,30]. This changes the temperature profile during and after deposition which also affects the polymer viscosity and bonding process between printed tracks. To successfully implement discontinuous fibres with a length above the critical fibre length as reinforcement in 3D printing filament, a matrix with good intrinsic processing characteristics is preferred.

A material selection process was performed to find the most suitable matrices for fibre reinforced 3D printing by collecting information from material databases and literature. The goal was to find matrix systems that will aid the development of a 3D printing filament with discontinuous fibres. Therefore, good processability of the polymer was considered most important. Several selection criteria were identified and used, from general properties to thermal and processing properties. Table 1 shows the full comparison of different plastics. Below, the selection criteria are discussed:

- From a product engineering perspective, the cost, density, stiffness and strength were considered. A lower density results in a lower overall weight and lower material costs are beneficial to reduce development costs and final filament costs. As the mechanical properties of the finite product are mainly driven by the reinforcement fibres, the strength and stiffness of the matrix itself are less important. Mechanical properties such as fracture toughness or brittleness of the polymer may be considered for specific performance requirements but for this initial selection these properties are left out.
- The thermal properties of the matrix are important to consider in the material trade-off as they influence the FFF process. Ideally, the matrix material should have a low processing temperature to reduce energy costs and a high usage temperature, however, these are conflicting requirements. For the trade-off, the melt temperature could not be used as amorphous polymers do not have a distinct melt temperature, and instead increasingly soften above their glass transition temperature *T*_g_. To compare both semi-crystalline and amorphous polymers, the well-documented processing temperature is used. This is a relevant material characteristic which gives a good comparison of the average processing temperature required for different types of polymers.
- The glass transition temperature is a property of both semi-crystalline and amorphous polymers that relates to softening of the polymer. Above the glass transition temperature polymers lose their rigidity are not suitable for structural use.
- The specific heat capacity and thermal conductivity are included in the trade-off as they influence the heating and associated softening of the material. If the material has a high specific heat capacity and high thermal conductivity, more heat initially must be added to soften the polymer. The benefit of this is that more heat can be distributed after deposition between the printed tracks to improve the polymer sintering process [31]. Having a high specific heat capacity and thermal conductivity is therefore beneficial to the FFF process.
- The coefficient of thermal expansion (CTE) relates to the amount of shrinking as the material cools. This can create residual stresses in the 3D printed part and therefore a low CTE is preferred.
- The linear shrinkage expressed as % of the material from melt processing conditions to final cooled part is compared as a metric for minimising residual stresses. This is different than the CTE as other effects (such as crystallisation) may influence shrinkage [32].
- The crystallinity of the polymer is an important parameter for 3D printing that influences the melt behaviour of the material. FFF works best with polymers that are amorphous, despite their lower mechanical performance, as they have no distinct melting point and increasingly soften (lower viscosity) with increasing temperature. This means the polymer sintering process happens over a longer time when cooling down [31].
- The polymer sintering process is related to material flow and the reptation time, which is the time required to create a fully welded, entangled interface. Reptation theory predicts that the reptation time is dependent on temperature and the molecular mass of the polymer. Material flow is related to viscosity, which is a function of temperature and strain rate (process dependent) and the molecular weight as well. These properties cannot be readily compared between polymers, but a simplification is possible to obtain an alternative comparison that is related to these properties. The average moulding pressure is a well-documented property that depends on the processing temperature, viscosity and molecular weight of the polymer. A higher moulding pressure means the polymer, at its processing conditions, flows less easily and thus would have a higher viscosity and longer reptation time. Although no external pressure is applied in the FFF process, the extrusion process is pressure driven and the moulding pressure gives a relative comparison between polymer viscosities which is confirmed later by rheological testing of the candidate polymers.
- The last set of properties that are compared are the printing performance and the interfacial properties with carbon fibres. Some materials already have proven to be processable by FFF while others are less commonly used. In the development of an improved fibre reinforced 3D printing filament, a successful printable material is preferred. Likewise, the interfacial properties with carbon fibres plays an important role to maximise the reinforcing effect of the fibres.

The properties discussed above are compared for sixteen thermoplastic matrices, ranging from commodity polymers such as polypropylene and polyamides to high end polymers such as polyethyleneimine (PEI) and polyether ether ketone (PEEK). Common 3D printing materials were also considered such as acrylonitrile butadiene styrene (ABS), polylactic acid (PLA) and polyethylene terephthalate glycol (PETG). Each of these polymers is now considered in turn, highlighting key aspects that affect their use for improved fibre reinforced 3D printing filaments:

- PLA has the lowest thermal conductivity and specific heat capacity from the common 3D printing polymers, but exhibits good processing temperatures and has a low shrinkage [33]. PLA is a semi-crystalline material and one of the most used 3D printing thermoplastic filaments on the commercial market [4]. The ease with which this material can be printed can be attributed in part to its low shrinkage and good rheological behaviour over a wide temperature range, as seen from its relatively low molding pressure.
- ABS is another widely used thermoplastic filament which has better thermal conductivity properties than PLA, but it is expected to have a higher viscosity which negatively affects bonding [33]. ABS, however, is an amorphous polymer which is advantageous for 3D printing [8,31]. It is known that ABS is best printed in a closed, heated environment as it is sensitive to temperature variations and warping as it has a relatively high CTE [45].
- PETG shows very similar properties as ABS, with a lower viscosity and lower shrinkage which explains its growing popularity as a 3D printing feedstock in recent years [33].
- PA, or commonly known as Nylon, has different grades (PA-66 and PA-6, PA-11, PA-12, etc.). In general, PA shows average properties in all criteria [33]. It has good resistance against oils and common solvents and has good impact resistance and low friction properties. It is mainly used in the transportation sector for non-structural and electronics parts and it is also common in the consumer goods sector. It is known to degrade at higher temperatures and to absorb moisture [46]. It less commonly used as a 3D printing material than ABS, PETG or PLA.
- Polypropylene (PP) is a much used material for general plastic parts and moulding as it is cheap, and resistant to heat and impacts [46]. Despite having a low expected processing viscosity, polypropylene has a relatively high shrinkage and is known to warp during 3D printing. Moreover, the interfacial properties with carbon fibres are expected to be low [47].
- Low density polyethylene (LDPE) and high density polyethylene (HDPE) are two low cost plastic materials that have lower mechanical properties than PP. They are used for cheap consumables such as plastic bags and bottles. They have a high crystallinity and print trials have shown they are hard to print [48]. Another downside of PE are the lower interfacial properties with carbon fibres expected from their molecular structure.
- Polycaprolactone (PCL) is a bio-degradable polymer with a very low melt temperature. Despite the good processing properties such as low viscosity and shrinkage, the low melt temperature of PCL (~55 °C) makes processing harder as solidification increases processing times [33]. It also has a very low T_g_ and low mechanical properties and therefore is not commonly used for structural parts.
- Polycarbonate (PC) has better mechanical properties than the other commodity polymers and is known for its high impact strength [46]. It shows overall good properties for 3D printing. It has a relatively high processing temperature and a high viscosity, and it is an amorphous polymer which is advantageous for 3D printing.
- Polyvinylchloride (PVC) is another low-cost polymer with a lower processing temperature than PC. It has a high viscosity but low shrinkage [33]. They are mainly used for plastic piping for several applications. Processing of PVC is harder as it release toxins when heated and has several health and safety issues [46].
- Polybutylene terephthalate (PBT) is a semi-crystalline engineering thermoplastic with relatively low cost. It has a relatively high shrinkage but a low expected viscosity [33]. It is often added to filaments to improve the printing quality but is not currently available on the market as filament.
- Polyetherimide (PEI) is common high-end 3D printing material also known as Ultem [49,50]. It is an amorphous polymer with a very low shrinkage and good interfacial properties with carbon fibre [33]. It has a relatively high processing temperature and high viscosity, but additives may be used to improve the flow properties [49,50].
- Polyether ether ketone (PEEK) is a high-performance engineering thermoplastic used in structural applications [46]. Carbon fibre pre-preg tape are available to be used for autoclave processing and recently have been used in Laser Assisted Automated Tape Placement for direct manufacturing of aerospace grade parts [51,52,53]. It has been used in both SLS and FFF processes [32,54,55]. The material trade-off shows it has a high processing temperature, high viscosity, and a higher shrinkage than PEI which makes it less suitable for FFF 3D printing applications [33].
- Polyphenylene sulphide (PPS) is a cheaper high-performance semi-crystalline thermoplastic. It has good mechanical properties and due to its low viscosity it is a preferred choice for high performance carbon fibre thermoplastic composites [56]. For 3D printing applications it has the disadvantage that it has a relatively high processing temperature and shrinkage compared to other materials.
- Polysulfone (PSU) and polyether sulfone (PESU) are relatively new high performance amorphous polymers used in 3D printing [57]. They have a high glass transition temperature and can be used up to 250 °C [46,58] with good mechanical properties. The downside is a relatively high viscosity and processing temperature but low shrinkage.

A material selection was performed using weighted scores for the different criteria. The aim was to find matrices which are best for carbon fibre reinforced FFF, and as such the criteria relating to processability were more important than the performance-based criteria at this stage. The best candidate matrices were used to manufacture ADFRCs within the available lab facilities. This meant the processing temperature and costs were important from a practical point of view, and for processing the moulding pressure (directly related to the viscosity of the material as will be shown in Section 2.2) was important. All other properties had equal weighing except for the density, crystallinity and strength/stiffness which had a lower importance for this study as they relate more to the performance of the final composite, while the focus was on FFF processing. The final trade-off with the criteria, weightings and scores are shown in Table 2.

The most suitable polymer for 3D printing from this selection is ABS, followed by PETG and PLA. This is not necessarily a surprising result, as these are the three most popular 3D printing filaments currently on the market, although it was also not a forgone conclusion given the consideration of criteria and weightings driven by use of fibres within the FFF process. PBT also scores highly, but it is not commonly used as 3D printing feedstock and so availability may be an issue in the short to medium term. PCL has good temperature and moulding pressure characteristics that gives it a good score for 3D printing, despite its low mechanical properties and low usage temperature. The polysulfones (PSU, PESU) scored high and are indeed an upcoming 3D printing material, especially when temperature constraints would be removed. Polyamide was ranked 8th in this study, with overall medium properties. LDPE and PP have better processing temperature and expected lower viscosities than PA, but they have lower thermal properties and low interfacial properties with carbon fibre which makes them less attractive for fibre reinforced 3D printing feedstock. PC and PEI have good mechanical properties and good thermal properties but require higher processing temperatures and moulding pressures which makes them less attractive to use in the filament development phase. The same holds for PPS and PEEK. PVC is attractive because it has a low processing temperature but scores low on the expected viscosity and interfacial properties. Finally, HDPE is the least attractive for 3D printing in this trade off as it is a crystalline material which has relatively high processing temperature and expected high viscosity.

**Figure 4:**
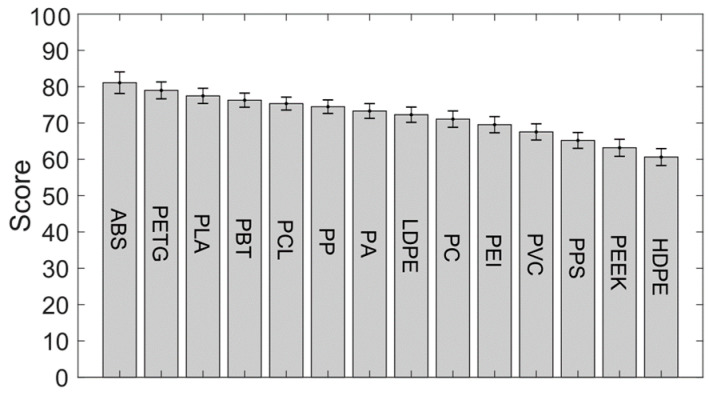
Sensitivity study on the ranking of the different polymers.

A sensitivity study was performed on the selection shown in Table 2, where the importance ratios and scores were changed randomly by ±2% and ±1 respectively over multiple runs (>1000) to see how sensitive the polymer rankings were to variance in the scores. The results are shown in Figure 4, which shows the ranking order based on the average scores across the sensitivity study along with the standard deviation of each polymer’s score. Here we see the top five choices are the same as in the single study shown above. The gradual drop in scores and the overlaps seen within the variance ranges of adjacent polymers implies that it would be prudent to consider more than just the top ranked choice.

Based on this study, ABS, PETG, PLA were chosen for further consideration as they are the three best performing matrices from the selection. PA, while not a very strong performer in this study, will also be considered moving forward as it provides a useful benchmark as the commercially available MarkForged continuous fibre filament is PA-based [59].

### 2.2. Matrix Analysis

**Figure 5:**
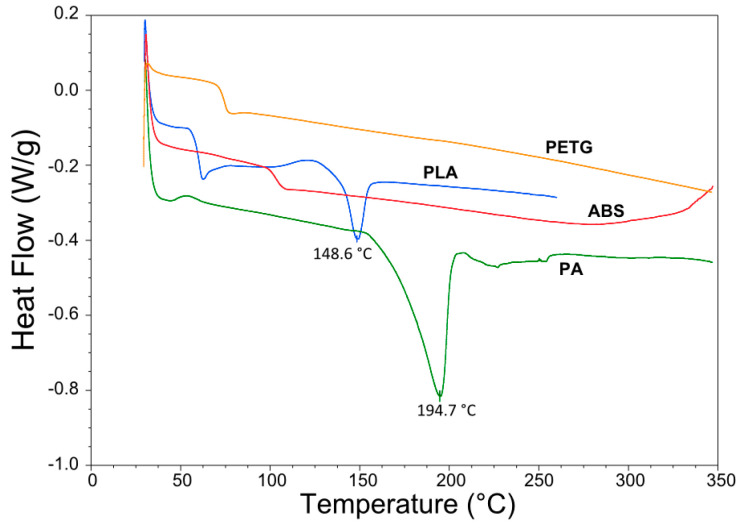
Digital scanning calorimetry (DSC) results of PA, PLA, PETG and ABS.

The selected polymers were characterized by digital scanning calorimetry (DSC), thermo-gravimetric analysis (TGA) and shear rheology. The four different polymers were acquired as 3D printing filament from 3D4Makers [60] and MarkForged [59]. DSC testing was performed on ~10 mg matrix samples using a Q200 by TA Instruments (New Castle, DE, USA) with an inert nitrogen atmosphere and a temperature ramp of 10 °C/min. The results are shown in Figure 5 which show the melt temperatures of the semi-crystalline polymers PA and PLA, being 148.6 °C and 194.7 °C. The amorphous polymers ABS and PETG do not melt and no endothermic peaks show up in the DSC curves for these two polymers.

**Figure 6:**
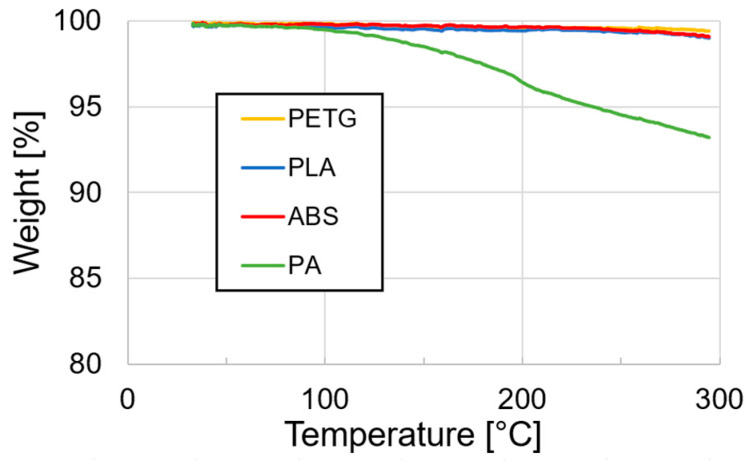
TGA data of the four different candidate polymers.

TGA was performed on an STA 449F3 system (Netzsch, Seld, Germany) under an oxidative atmosphere to identify whether the polymer exhibits any mass loss at higher temperature which may indicate degradation. The samples weighed between 8–10 mg and were heated to 300 °C at a rate of 10 °C/min, with a standard air atmosphere and a gas flow rate of 50 mL/min. The results are shown in Figure 6. From the four candidate polymers, only PA showed a mass loss starting from roughly 100 °C and at 300 °C lost 7% of its mass. This indicates possible degradation at higher temperature as expected for PA and/or may be related to water absorption of PA which it is prone to do [61]. The other candidate polymers (ABS, PETG, PLA) lost less than 1% of their mass at the end of the test.

**Figure 7:**
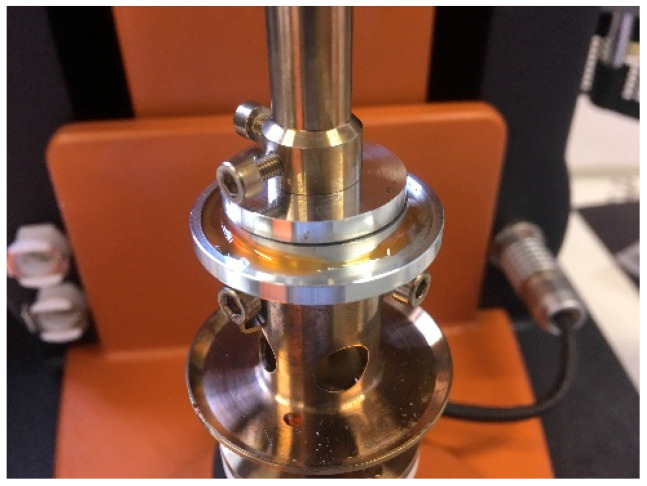
Shear rheology sample preparation showing the excess material and 0.6 mm gap.

Lastly, rheological testing was performed on the candidate polymers. A TA Instruments (New Castle, DE, USA) Discovery HR30 parallel plate rotational rheometer was used, with a 25 mm diameter plates and a shearing gap of 0.6 mm. The samples were prepared as 3D printed disks with a diameter of 20 mm and a thickness of 1.1 mm, such that the entire gap was filled when the parallel plates were lowered to the shearing gap of 0.6 mm. Any excess material was removed after squeezing of the disks as shown in Figure 7. Oscillatory measurements were performed at 1 rad/s, 10 rad/s and 100 rad/s at temperatures up to 300 °C and using a standard atmosphere.

**Figure 8:**
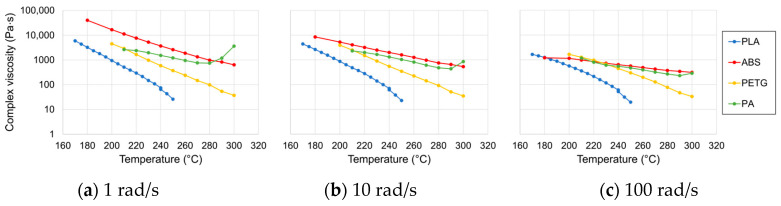
Complex viscosity measurements at different strain rates for the candidate polymers.

Figure 8 shows the complex viscosity of the candidate polymers. All samples clearly show a reduction in viscosity for increasing temperatures as expected. Shear thinning can also be observed, which was most pronounced for ABS as the viscosity reduced by an order of magnitude for a strain rate of 100 rad/s compared to 1 rad/s. For the other polymers, the effect of shear thinning was less pronounced. Comparing the difference in viscosities to the predicted trends from the documented moulding pressures, one can see that ABS (113.5 MPa) indeed has the highest viscosity, followed by PA (89.42 MPa) and with PLA (77.5 MPa) and PETG (72.44 MPa) having a similar viscosity range. This confirms that the moulding pressure can be used as a relative comparison for the polymer viscosity.

### 2.3. Fibre Reinforcement

The aim of this work is to make a thermoplastic composite tape with aligned discontinuous fibres where the full strength of carbon fibres is reached. This requires the fibre length to be above a critical length such that fibre failure occurs before interfacial failure. The critical fibre length is dependent on the fibre type and surface treatment and the matrix type. A simple approximation of the load transfer can be made with 1D shear lag theory, assuming load transfer to the fibre only takes place through the interface [12]. This gives Equation (1) for the critical fibre length *l*_c_ as a function of fibre strength *σ*_fu_, fibre diameter *d*_f_ and interfacial shear strength τ_i_ [13]. More advanced models have been developed, taking into account the relative deformation of the fibres and resin to better predict the shear stress transfer, but the concept of a critical fibre length for discontinuous fibres is well understood [12,14,62]:

(1) $$l_{c}=\frac{\sigma_{fu}}{2\tau_{i}}d_{f}$$

For PA and PLA, the interfacial shear strength (IFSS) was found in literature [34,35]. The critical fibre length was estimated using Equation (1) and found to be 0.78 mm and 0.8–1.38 mm, respectively. This provides a rough estimate of the critical fibre length, which will be dependent on the exact polymer composition, fibre type and sizing.

It was chosen to use 3 mm carbon fibres to ensure the critical length was exceeded. Toho Tenax C124 fibres were used which have been used with the HiPerDiF fibre alignment method before [15,17,63,64]. These fibres have a water-soluble sizing which allows them to disperse well in water which aids the HiPerDiF fibre alignment process. Because the sizing is water-soluble, some loss of the sizing can be expected during the HiPerDiF process, however this is expected to be minimal as the fibres spent little time (<5 min) in suspension. The properties of the fibres are shown in Table 3 below.

### 2.4. Fibre Alignment Process

**Figure 9:**
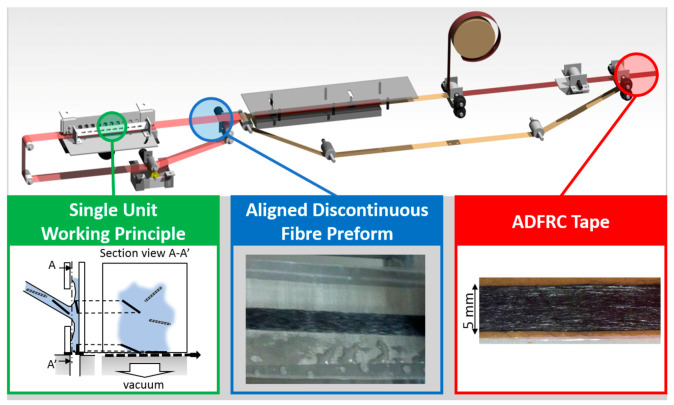
Overview of the HiPerDiF fibre alignment method showing the working principle and dry fibre preform output.

The HiPerDiF fibre alignment process was used to create aligned 3 mm fibre preforms, which were then coupled with a thermoplastic matrix. Highly aligned fibre preforms allow for a higher packing ratio and higher fibre volume fractions, leading to an increase in mechanical properties. Figure 9 shows the working principle of the HiPerDiF process. Fibres are suspended in water and sprayed between two parallel plates (alignment head) with a small gap between them. Due to a sudden momentum change at the impact with the plate the fibres align parallel to the plates and fall between them onto a moving mesh conveyor belt. Vacuum is applied underneath the belt to remove the water and after drying a tape of discontinuous fibres is obtained [15].

By controlling the flow rate and belt speed, the weight of the fibre preforms can be changed. For this work, fibre preform tapes with a width of 5 mm and aerial weight of 60 g/m^2^ were prepared.

### 2.5. Composite Manufacture

**Figure 10:**
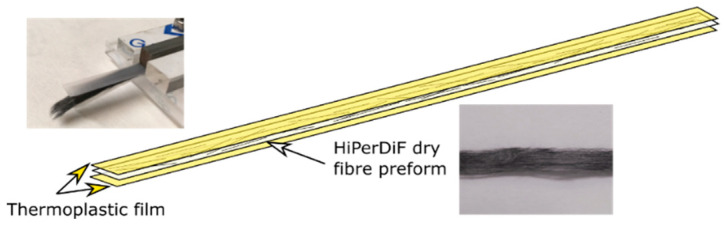
Preform before consolidation showing aligned 3 mm fibres sandwiches between polymer films.

**Figure 11:**
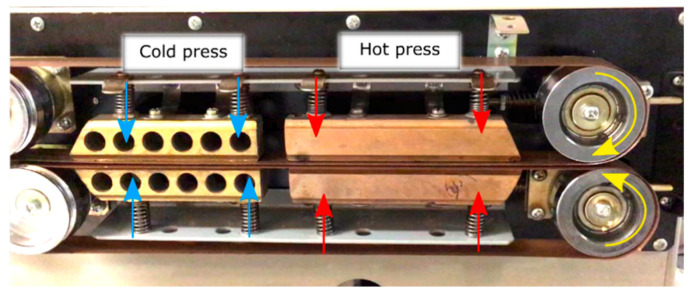
Pre-pregging module for consolidation of the fibres with the matrix.

The four candidate polymers were used as matrix for the ADFRCs; ABS, PLA, PETG and PA. These materials were acquired as 3D printing filament from 3D4Makers [60] and MarkForged [59] and 3D printed into films with a 0.125 mm thickness for film impregnation. For the consolidation process, film impregnation was used where a sandwich of a single aligned dry fibre preform between two polymeric matrix films was fed through a custom double-belt hot press, as shown in Figure 10 and Figure 11.

In the double belt hot press, pressure and heat were applied to consolidate the fibres and the matrix into a composite material. The consolidation module consists of two glass fibre reinforced PTFE belts, a hot press and a cold press. Darcy’s law gives a relation between pressure *p*, time *t*, viscosity *η*, permeability *K* and resin flow rate for consolidation [65]. Darcy’s law can be integrated to yield Equation (2) and give a direct relationship between the time *t* and the flow length *L* with different processing conditions:

(2) $$t=\frac{L^{2}\eta }{2K\Delta p}$$

The permeability of the fibre bed depends on the packing structure, volume fraction and fibre dimensions. The permeability is approximated using Gebart’s derivation for the perpendicular permeability of uni-directional reinforcement [66]. Quadratic fibre packing was assumed as a conservative value for the permeability, which was calculated using Equation (3), where *R_f_* is the fibre diameter. Although the fibres may be not be perfectly aligned and have different packing structures, any misaligned fibres will make a more porous preform that increases permeability such that the conservative case is considered. The permeability was found to be 7.9 × 10^−12^ m^2^ using a fibre volume fraction of 12.5% and fibre radius of 3.5 μm:

(3) $$K_{⊥,quad}=\frac{16}{9\pi \surd 2}{\left(\sqrt{\frac{V_{f,max}}{V_{f}}}-1\right)}^{\frac{5}{2}}R_{f}^{2}\;\mathrm{with}\;V_{f,max}=\frac{\pi }{4}$$

A pressure mapping system (Tekscan, South Boston, MA, USA) was used to determine the applied consolidation pressure. Because the hot plates are spring loaded, the total compressive force depends on the thickness of the material. A thin film tactile pressure sensor (0.1 mm) was enveloped between two sheets of paper to obtain a total thickness of 0.2 mm for force measurements, equivalent to the average ADFRC tape thickness. The total compressive force was 49.1 N over the entire hot plate area (100 mm × 10 mm). During consolidation, that force is applied onto the tape which has a width of 5 mm, and this results in an average consolidation pressure of 0.098 N/mm^2^ or roughly 1 bar.

The measured tape thickness was ~0.2 mm and this correlates to a processing time of 25 s for a viscosity of 1000 Pa·s (rough average of all polymers from rheological testing at low strain rate) and 1 bar of consolidation pressure. The belt speed was changed to have a 25 s consolidation stage to obtain good impregnation for all specimens which were visually inspected. The dry fibre preforms, polymer films and offcuts were weighed before and directly after processing, from which the fibre volume fraction *V*_f_ was estimated. After processing, the ADFRCs were trimmed to 100 mm length and 4 mm width to create uniform tensile test specimens.

Different processing temperatures were investigated for the different matrix systems as shown in Table 4. These temperatures were chosen to be the lower and upper bounds of the recommended processing temperatures according to CES Edupak [33]. The PA upper processing temperature was based on the manufacturer recommended printing temperature of 260 °C [59]. For the lower processing temperature, a value of 200 °C was selected to minimise potential degradation of the matrix.

### 2.6. Mechanical Testing

**Figure 12:**
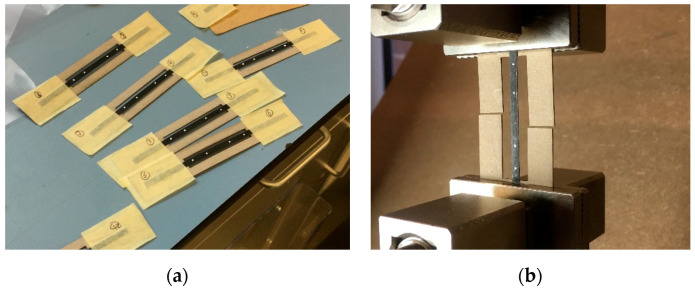
Tensile test samples during (**a**) preparation and (**b**) loaded in tensile test machine.

The ASTM D3030 test standard for tensile properties of polymer matrix composites was used as reference for tensile testing. The samples were smaller than recommended (100 mm × 4 mm × 0.2 mm) as they were limited in width by the HiPerDiF fibre alignment process. The thickness and width were measured at three points for each sample and for each configuration, three samples were tested. The ADFRCs were taped to a paper tab as shown in Figure 12 and clamped between two mechanical grips of an electromechanical Shimadzu test machine equipped with a 1 kN load cell. The specimen was loaded at the recommended displacement rate of 1 mm/min and a video extensometer by iMetrum was used to measure the strain during testing.

## 3. Results

This section may be divided by subheadings. It should provide a concise and precise description of the experimental results, their interpretation as well as the experimental conclusions that can be drawn.

### 3.1. Mechanical Testing

**Figure 13:**
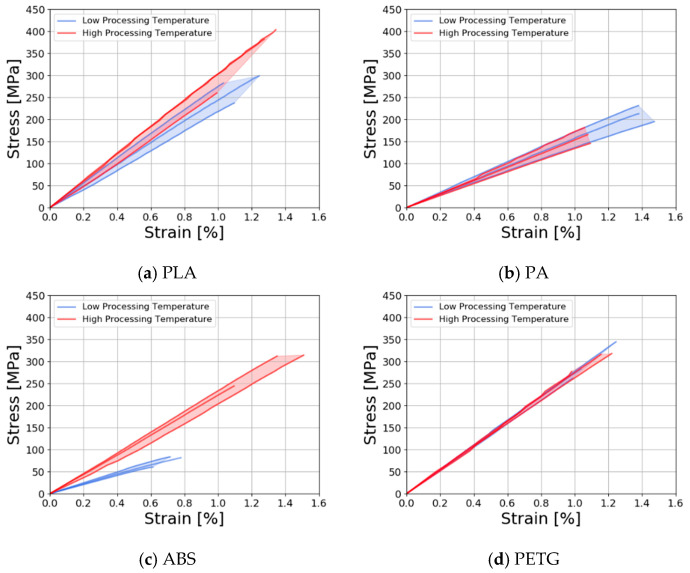
Stress-strain curves of ADFRCs at low processing temperature and high processing temperature with (**a**) PLA; (**b**) PA; (**c**) ABS; (**d**) PETG matrix.

The tensile response of the four different ADFRCs can be seen below in Figure 13, where all stress-strain curves are shown for the different specimens. In general, all specimens showed a linear stress-strain curve until failure. This shows the reinforcing effect of the discontinuous fibres, as plastic deformation was prevented, and the maximum elongation was limited to about 1.1%. Overall, the best performing specimens had a strength of around 300 MPa and a stiffness on the order of 23 GPa, which included the PETG, PLA and ABS specimens. 

The PLA specimens (Figure 13a) showed a small increase in strength and stiffness for the higher processing temperatures as consolidation may have improved due to the lower viscosity during processing. The ABS specimens (Figure 13b) showed a large increase in performance at higher processing temperatures, which was caused by poor consolidation at the low processing temperatures. At 177 °C, ABS has the highest viscosity of the polymers tested which meant the polymer did not fully impregnate the specimen, which was confirmed via visual inspection. For the PA specimens (Figure 13) the strength decreased somewhat at higher processing temperatures which may be caused by degradation of the matrix as confirmed by TGA testing presented in Section 2.2. The PETG specimens (Figure 13d) showed the lowest variation and little change between processing temperatures. Table 5 shows average test results with coefficient of variance (CoV) for the different tensile properties.

**Figure 14:**
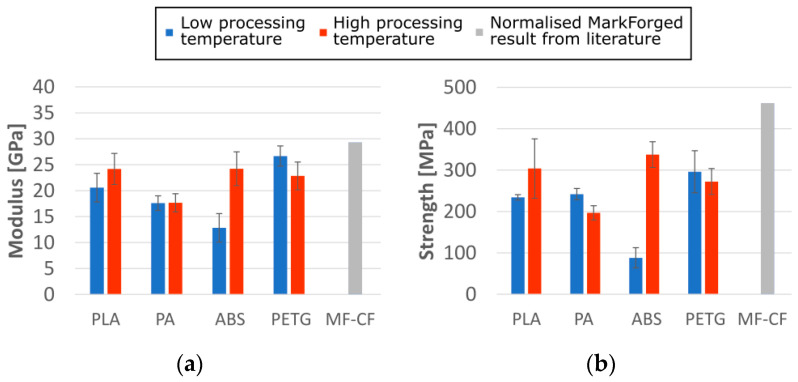
Tensile test results for (**a**) modulus and (**b**) strength of ADFRCs processed at different temperatures as measured compared to MarkForged continuous fibre (MF-CF) printed parts normalized by ratio to a V_f_ of 12.5% [8].

A comparison of the strength of stiffness of each ADFRCs is shown in Figure 14, which also shows the standard deviation of each three specimens tested. The increase in performance of the ABS specimens at higher processing temperatures can clearly be seen here, as well as the lower overall performance of the PA specimens. For reference, the tensile properties of the MarkForged continuous fibre PA filament are also shown as reported in [8], normalised from a *V*_f_ of 20% to a *V*_f_ of 12.5% by ratio.

### 3.2. Micrographs

**Figure 15:**
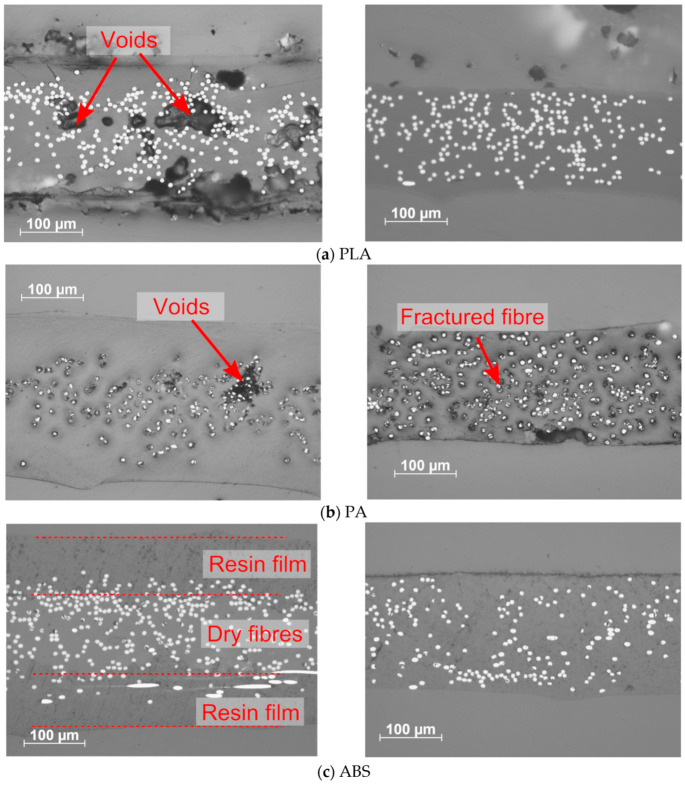

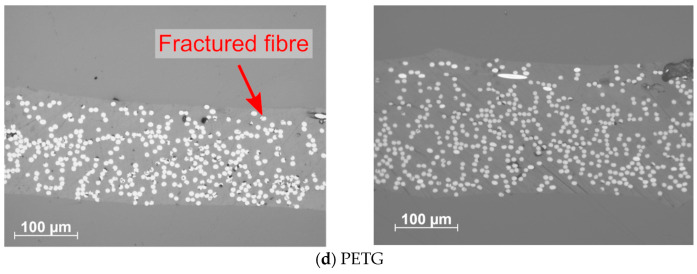
Optical micrographs of different ADFRPs, left low processing temperatures and right high processing temperatures: (**a**) PLA (**b**)PA (**c**) ABS (**d**) PETG.

Two different micrograph studies were performed, using optical microscopy and scanning electron microscopy (SEM) for fracture surface analysis. Figure 15 shows representative cross sections of the four different ADFRCs at low and high processing temperatures. The PA ADFRCs show a dark region around the fibres which is attributed to polishing artefacts caused by the low modulus of PA and the poor adherence to the carbon fibres. For the other samples, few polishing artefacts were present around the ADFRCs, providing a clear image of their cross sections.

The PLA and ABS specimens showed a clear reduction in void content from a low processing temperature to a high processing temperature. The low temperature processed ABS specimen showed poor consolidation as two distinct resin films with dry fibres in the middle could be observed. For the PETG specimens, similar quality was observed for the low and high processing temperatures. Small darker regions near the fibres can be seen that show fibre fracture in the specimen, which is also present to some extent in the other specimens.

**Figure 16:**
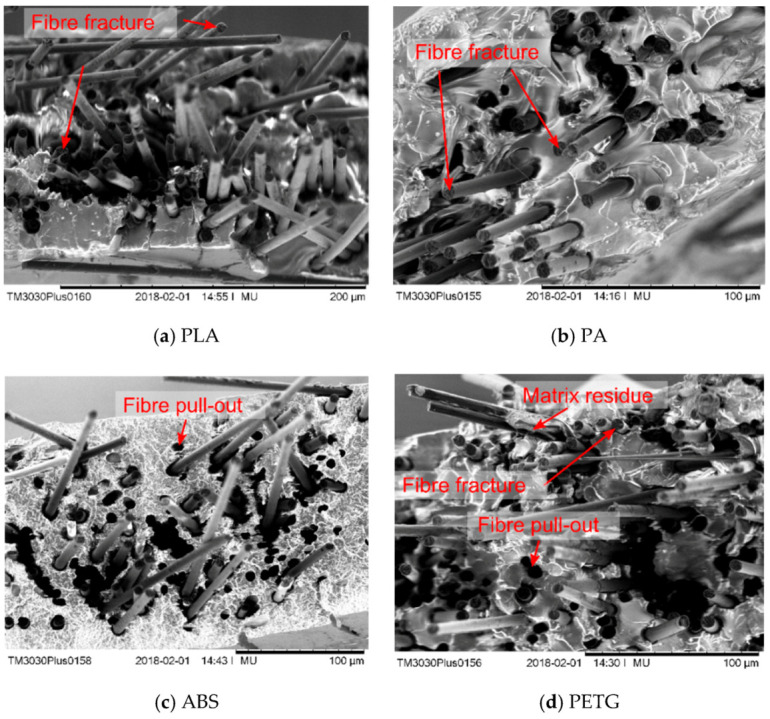
SEM micrographs of fracture surfaces of different ADFRCs, all for higher processing temperatures: (**a**) PLA (**b**) PA (**c**) ABS (**d**) PETG.

Figure 16 shows the fracture surfaces obtained from SEM of four different ADFRCs, all processed at higher processing temperatures. The PA specimen clearly shows more plastic deformation of the matrix compared to the other specimens. The serrated and rough fibre ends indicate fibre fracture occurred. Some fibre-pull out appears to be also present which as clear circular holes in the resin are visible. Overall, little matrix residue was found on the fibres which shows the fibre-matrix interface is poor.

## 4. Discussion

A matrix selection for fibre reinforced FFF was presented based on consideration of a wide range of criteria that influence the mechanical behaviour and process dynamics. In this work, the goal was to select polymers that are suitable for future fibre reinforced FFF processing and as such the processing temperatures and the polymer viscosity were more important at this stage than performance criteria such as crystallinity and strength/stiffness. A substitute for polymer viscosity was used (the documented average moulding pressure) which allowed comparison of different polymers. Rheological testing of the candidate polymers showed similar trends between their relative viscosities and documented moulding pressure, confirming this can be used as a relative comparison between polymers.

The study highlighted ABS, PLA, and PETG as the most promising polymers for future FFF use. Other polymers have been identified which can be suitable for fibre reinforced 3D printing such as PBT. This process may therefore be used for future identification of suitable 3D printing materials, where the criteria and weightings may be changed depending on temperature and cost restrictions and performance optimisation. The performance of the fibre reinforced thermoplastic composites was investigated by the manufacture of ADFRC tapes.

ADFRC tapes were manufactured using a custom-built consolidation module. A flow analysis through Darcy’s law indicated enough pressure was applied to impregnate the fibres within 25 s. However, some voids were found on the specimens and further improvement may be possible by increasing the pressure and/or time of consolidation. The flow of the polymer may also affect the fibre alignment as the fibres are not fixed in place. An improvement could be made by preventing any side flow through redesign of the consolidation station.

The reinforcing effect of the fibres was clearly present as the strength increased by a factor 7.5 from ~40 MPa for the pure polymer to 300 MPa of the ADFRCs. The measured average failure strength of 300 MPa was lower than the theoretical continuous fibre composite strength (~580 MPa) based on a rule of mixtures analysis of fibre strength and fibre volume fraction. The same holds for the predicted composite stiffness of roughly 30 GPa compared to the measured stiffness of ~23 GPa. This may be attributed to uneven fibre distribution, fibre misalignment and the discontinuous fibres not being fully loaded as explained by shear lag theory [67].

A better prediction of the ADFRC modulus is given by Equation (4), where the composite modulus *E_c_* is calculated as a function of the fibre modulus and content (*E_f_*, *V_f_*), matrix modulus and content (*E_m_*, *V_m_*) and the fibre misalignment factor *η*_0_ and fibre efficiency factor *η*_1_ [15]. The fibre efficiency factor *η*_1_ relates to the fibres not being completely loaded. It can be calculated using the analysis presented by Cox [62]. It is dependent on the fibre length *l_f_* and diameter *d_f_* and a constant *a* relating to the matrix modulus *E_m_*, fibre modulus *E_f_* and fibre volume content *V_f_* as shown in Equations (5) and (6). Substituting the known values for the carbon fibres and PLA matrix, with a *V_f_* of 12% gives *η*_1_ = 0.970:

(4) $$E_{c}=\eta_{0}\eta_{1}V_{f}E_{f}+V_{m}E_{m}$$

(5) $$\eta_{1}=1-\frac{\tan h\left(al_{f}/d_{f}\right)}{al_{f}/d_{f}}$$

(6) $$a=\sqrt{-\frac{3E_{m}}{2E_{f}ln(V_{f})}}$$

**Figure 17:**
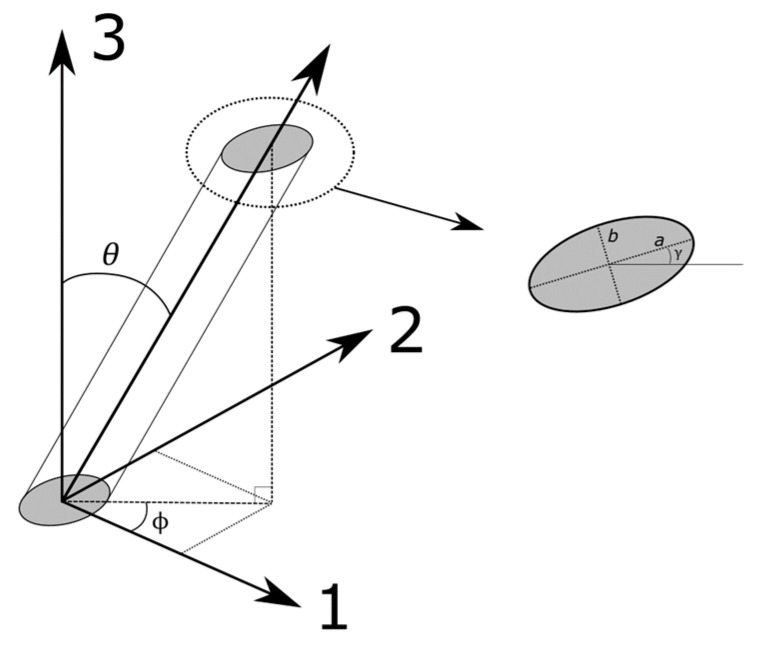
Definition of fibre orientation expressed as angles (θ,ϕ).

For a composite cross section, each fibre will be represented by an ellipse, and its major axis *a* and minor axis *b* will change direction and magnitude depending on the fibre orientation as shown in Figure 17. The fibre orientation angles θ and ϕ and can obtained using Equations (7) and (8), where θ is the in-plane angle the fibre makes with the 1–2 plane and ϕ is the out-of-plane angle.

(7) $$\theta =\cos^{-1}\left(b/a\right)$$

(8) ϕ=γ

**Figure 18:**
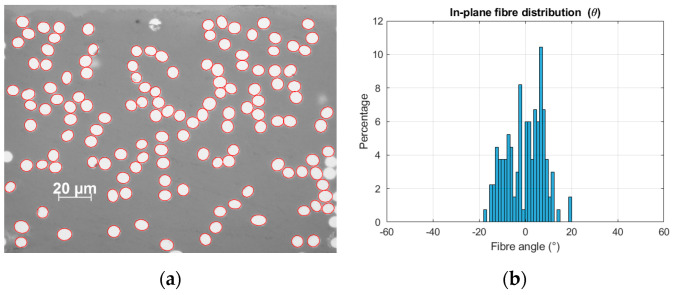
Fibre orientation analysis showing (**a**) ellipse fitting performed on PLA cross section and (**b**) resulting in-plane fibre distribution.

Ellipse fitting was performed via the ParticleSizer script for ImageJ [68]. Figure 18 shows the ellipse fitting for an example cross section of the PLA ADFRC with the accompanying in-plane fibre distribution. As the micrograph cross section can be misaligned with respect to the fibre tape, the mean fibre orientation was calculated and used to centre the fibre orientation distribution around 0°. The fibre orientations fall between ±20°, with 37% of the fibres bounded between ±5°, which is lower than expected from previous HiPerDiF results.

The fibre misalignment factor *η*_0_ is calculated using an analogue to classical laminate theory, by representing each fibre as a lamina with an angle *θ*. The modulus for each lamina can be calculated using Equation (9), where *E*_11_ is the composite modulus along the fibre direction, *E*_22_ is the composite modulus perpendicular to the fibre direction, *G*_12_ is the in-plane shear modulus and *ν*_12_ is the in-plane Poisson ratio. These properties are estimated by rule of mixtures using a *V_f_* of 12.5% and the fibre and matrix properties. The effective composite modulus E_c_^*^ with different fibre orientations can then be calculated using Equation (10), and the fibre misalignment factor is defined as the ratio between *E_c_* and *E_θ_* as shown in Equation (11):

(9) $$\frac{1}{E_{\theta }}=\frac{\cos^{4}\theta }{E_{11}}+\left(\frac{1}{G_{12}}-\frac{2\nu_{12}}{E_{11}}\right)\sin^{2}\theta \cos^{2}\theta +\frac{\sin^{4}\theta }{E_{22}}$$

(10) $$E_{c^{*}}=\overset{n}{\underset{k=1}{\sum }}\frac{E_{\theta ,k}}{n}$$

(11) $$\eta_{0}=\frac{E_{c}^{*}}{E_{\theta }}$$

The fibre misalignment factor *η*_0_ was found to be 0.874 and this results in a predicted stiffness of 25.36 GPa for the PLA sample, which is within one standard deviation from the best performing samples. An increase in mechanical performance may therefore be possible by increasing the level of fibre alignment.

The stress-strain curves exhibited a linear trend with a maximum strain of ~1.1% which indicates fibre fracture was obtained for these specimens. The SEM micrographs showed a combination of fibre fracture (serrated fibre ends), together with some fibre pull-out. One can see that little matrix residue was left on the fibres which indicated a generally poor interfacial adhesion between the thermoplastic matrices and the carbon fibres.

The use of fibres significantly beyond the critical length has in large part made up for deficiencies of the interface through the simple addition of interface area. In concurrent work, closed-loop recycling of thermoplastic matrix composites was investigated which showed an increase in strength over multiple recycling loops which shows an increase in strength may be possible by optimizing the fibre-matrix interface [16,17].

The ABS samples showed a large increase in mechanical properties at higher processing temperatures. This was confirmed by the poor impregnation of the ABS samples at lower temperatures as seen in Figure 15c. ABS was expected to be the best performing and processable material from the material selection and has the highest average stiffness of 25 GPa.

The PA matrix ADFCR clearly showed local ductile deformation of the matrix on the SEM micrograph and it showed the lowest performance of the four tested materials. This sample was fabricated to compare it to the MarkForged continuous fibre printer, which prints a carbon fibre—PA composite with a *V*_f_ of 20% and a tensile stiffness and strength of 50 GPa and 700 MPa respectively. The ADFRC PA composites in this study had a *V*_f_ of a 12.5% and showed a stiffness and strength of 17.3 GPa and 239 MPa. In this study, the strength and stiffness reduced at higher processing temperature which could be due to oxidation of the matrix as TGA analysis confirmed.

From the matrix selection process, PLA, PET and ABS were expected to perform better than PA as ADFRC matrix. The results confirm this, where the highest mechanical performance was obtained with PLA, PETG and ABS matrices with strength and stiffness in the order of 300 MPa and 23 GPa. The highest measured strength was 407 MPa and the highest measured modulus was 30.9 GPa for a PLA sample for a relatively low fibre content of 12.5%. This is a factor 8 increase in strength compared to available short fibre thermoplastic filament with similar matrices [5,6,69,70]. 

## 5. Conclusions

Aligned discontinuous fibre reinforced composites (ADFRCs) were investigated as a feedstock material for automated composites manufacture using extrusion based additive manufacturing. The hypothesized advantage of ADFRCs is that they can provide near continuous fibre composite performance but have better processing characteristics as the discontinuous fibres can be more freely deposited. Aligned 3 mm long carbon fibres were used which is well above the average reported critical fibre length of ~1 mm for carbon fibres in thermoplastic matrices. A matrix selection was performed based on the process characteristics of fused filament fabrication which yielded four candidate thermoplastic matrices (ABS, PLA, PETG and PA). 

An in-house developed continuous consolidation module was used to manufacture composite tape with a *V*_f_ of 12.5%, which were then subjected to a tensile test. Tensile stiffnesses and strengths up to 30 GPa and 400 MPa respectively were recorded with failure strains in the order of 1.1%. Compared to currently available short fibre thermoplastic filaments, the strength of the ADFRCs was an order of magnitude higher. Fracture surface analysis revealed a combination of fibre fracture and fibre pull out. Little matrix residue was found on the fibres which indicates the interfacial properties may be improved. Overall, the measured strength was lower than theoretically predicted. This may be attributed to an uneven fibre distribution, misalignment and incomplete load-transfer, and therefore better understanding of the flow properties of the matrices and fibre-matrix interface may improve consolidation and the mechanical properties. 

This work demonstrated the performance of ADFRCs and that they can compete with their continuous fibre counterpart. Future work focuses on investigating the flow properties of the ADFRCs and how the fibres behave in a thermoplastic melt. This should allow extrusion based additive manufacture of these ADFRCs, enabling a low-cost manufacturing method for high performance materials.

## Figures and Tables

**Table 1:** Comparison chart for suitability of different polymer for fibre reinforced 3D printing.

	Explanation	PA	PLA	ABS	PETG	PP	PCL	PC	LDPE	HDPE	PVC	PBT	PEI	PEEK	PPS	PSU	PESU
**Other names**	Commercial and/or full name	Polyamide (Nylon)	Poly lactic acid	Acrylonitrile butadiene styrene	Poly ethylene terephthalate glycol	Poly propylene	Poly caprolactone	Poly carbonate	Low-density poly ethylene	High-density poly ethylene	Poly vinyl chlorid	Poly butylene terephthalate	Polyetherimide (Ultem)	Poly ether ether ketone	Poly phenylene sulfide	Polysulfone	Polyether-sulfone
**Structure**	Can be important to derive adhesion to fibres	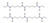	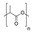	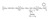	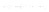	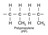	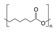	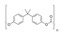	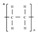	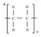	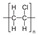	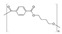	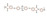	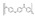	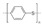	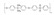	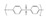
**Strength /stiffness**	Mechanical properties	0.94–1.18 GPa 38.6–48.2 MPa [33]	2.3–2.6 GPa 38–68 MPa [33]	2–2.9 GPa 29.6–44.1 MPa [33]	2.01–2.11 GPa 47.9–52.9 MPa [33]	1.34-1.59 GPa 32.9–36.4 MPa [33]	0.39–0.44 GPa 21.1–38.5 MPa [33]	2.32–2.44 GPa 59.1–65.2 MPa [33]	0.17–0.28 GPa 8.96–14.5MPa [33]	1.07–1.09 GPa 26.2–31 MPa [33]	2.48–3.3 GPa 41.4–52.7 MPa [33]	1.93–3 GPa 56.5–60 MPa [33]	2.89–3.04 GPa 73.5–81.1 MPa [33]	3.79–3.95 GPa 87–95 MPa [33]	3.23–3.39 GPa 64–67.2 MPa [33]	2.62–2.76 GPa 94.4–104 MPa [33]	2.76–2.9 GPa 85.4–94.1 MPa [33]
**Density**	Density	1060–1080 kg/m^3^ [33]	1110–1210 kg/m^3^ [33]	1020–1080 kg/m^3^ [33]	1260–1280 kg/m^3^ [33]	899–908 kg/m^3^ [33]	1140–1150 kg/m^3^ [33]	1190–1210 kg/m^3^ [33]	914–932 kg/m^3^ [33]	952–965 kg/m^3^ [33]	1300–1490 kg/m^3^ [33]	1300–1380 kg/m^3^ [33]	1260–1280 kg/m^3^ [33]	1300–1320 kg/m^3^ [33]	1340–1360 kg/m^3^ [33]	1230–1250 kg/m^3^ [33]	1360–1380 kg/m^3^ [33]
**Costs**	Lower cost better	2.35–2.53 GBP/kg [33]	2.34–3.01 GBP/kg [33]	1.82–2.15 GBP/kg [33]	1.98–2.06 GBP/kg [1]	1.1–1.14 GBP/kg [33]	5.73–8.32 GBP/kg [33]	2.57–2.75 GBP/kg [33]	1.32–1.35 GBP/kg [33]	1.22–1.25 GBP/kg [33]	1.06–1.21 GBP/kg [33]	1.98–2.06 GBP/kg [33]	13.4 GBP/kg [33]	75 GBP/kg [33]	4.83–5.23 GBP/kg [33]	7.53–10.9 GBP/kg [33]	8.73–9.26 GBP/kg [33]
**Glass transition temperature**	High glass transition temperature for usage	~60 °C [33]	52–82.6 °C [33]	88–120 °C [33]	81–91 °C [33]	−14, −6 °C [33]	−72, −59 °C [33]	142–158 °C [33]	−125, −90 °C [33]	−125, −90 °C [33]	80–88 °C [33]	22–43 °C [33]	215–217 °C [33]	143–157 °C [33]	81–97 °C [33]	186–192 °C [33]	210–235 °C [33]
**Processing temperature**	Lower processing temperature is easier/cheaper	220–327 °C [33]	170–210 °C [33]	177–260 °C [33]	249–288 °C [33]	203–250 °C [33]	106–133 °C [33]	205–298 °C [33]	121–232 °C [33]	177–274 °C [33]	177–199 °C [33]	184–274 °C [33]	309–430 °C [33]	349–399 °C [33]	257–338 °C [33]	273–360 °C [33]	295–391 °C [33]
**Coefficient thermal expansion (CTE)**	Low thermal expansion to prevent warpage	141–147 μstrain/°C [33]	126–145 μstrain/°C [33]	128–234 μstrain/°C [33]	120–123 μstrain/°C [33]	81.1–109 μstrain/°C [33]	158–172 μstrain/°C [33]	120–125 μstrain/°C [33]	180–396 μstrain/°C [33]	106–198 μstrain°C [33]	90–180 μstrain/°C [33]	108–171 μstrain/°C [33]	84.6–101 μstrain/°C [33]	50–60 μstrain/°C [33]	48.6–88.2 μstrain/°C [33]	54.7–56.9 μstrain/°C [33]	54.7–56.9 μstrain/°C [33]
**Thermal conductivity**	High thermal conductivity, easy to heat up and redistribute heat for polymer sintering	0.24–0.32 W/m°C [33]	0.13–0.16 W/m°C [33]	0.266–0.235 W/m°C [33]	0.257–0.267 W/m°C [33]	0.205–0.214 W/m°C [33]	0.17–0.18 Wm/°C [33]	0.193–0.218 W/m°C [33]	0.322–0.348 W/m°C [33]	0.461–0.502 W/m°C [33]	0.147–0.209 W/m°C [33]	0.274–0.285 W/m°C [33]	0.123–0.13 W/m°C [33]	0.24–0.26 Wm/°C [33]	0.23–0.29 Wm/°C [33]	0.277–0.288 Wm/°C [33]	0.291–0303 Wm/°C [33]
**Specific heat capacity**	Higher is better, more heat can be added in the system when extruding	1.65e3–1.71e3 J/kg°C [33]	1.18e3–1.21e3 J/kg°C [33]	1.39e3–1.41e3 J/kg°C [33]	1.47e3–1.53e3 J/kg°C [33]	1.66e3–1.7e3 J/kg°C [33]	1.42e3–1.5e3 J/kg°C [33]	1.15e3–1.25e3 J/kg°C [33]	1.84e3–1.92e3 J/kg°C [33]	1.75e3–1.81e3 J/kg°C [33]	1e3–1.1e3 J/kg°C [33]	1.42e3–1.48e3 J/kg°C [33]	1.47e3–1.53e3 J/kg°C [33]	1.34e3 J/kg°C [33]	1.41e3–1.47e3 J/kg°C [33]	1.5e3–1.53e3 J/kg°C [33]	1.4e3–1.45e3 J/kg°C [33]
**Crystallinity**	Lower is better, want to print viscous and better bonding [31]	Semi-crystalline	Semi-crystalline	Amorphous	Amorphous	Semi-crystalline	Semi-crystalline	Amorphous	Semi-crystalline	High level of crystallinity	Semi-crystalline	Semi-crystalline	Amorphous	Semi-crystalline	Semi-crystalline	Amorphous	Amorphous
**Shrinkage**	Low shrinkage from melt to prevent warpage	1.2-1.8 % [33]	0.3–0.4% [33]	0.4–0.7% [33]	0.2–0.5% [33]	1.4–1.95% [33]	0.15–0.7% [33]	0.5–0.7% [33]	1.5–5% [33]	1.5–4% [33]	0.2–0.6% [33]	0.9–2.2% [33]	0.5–0.7% [33]	1–1.21% [33]	0.6–1.4% [33]	0.5–0.7% [33]	0.7–1% [33]
**Molding pressure**	Used as it is related to viscosity	6.88–172 (89.44) MPa	55–100 (77.5) MPa	55–172 (113.5) MPa	6.88–138 (72.44) MPa	18–115 (66.5) MPa	50–52 (51) MPa	55–138 (96.5) MPa	34.3–103 (68.65) MPa	82–103 (92.5) MPa	68.8–275 (171.9) MPa	27.5–68.8 (48.15) MPa	69–138 (103.5) MPa	68.8–138 (103.4) MPa	13.8–20.6 (17.2) MPa	34.4–138 (86.2) MPa [33]	41.3–138 (89.7) MPa [33]
**Printing capability**	Check proven 3D printing capability	Higher printing temperature than PLA/ABS, needs to be dry.	Good printing characteristics.	Good printing characteristics.	Good printing characteristics.	Hard to print. Warping problems due to high crystallinity.	Seems good, multiple reports on 3D printing of PCL.	Printing demonstrated, quite high temperatures.	Can be used for printing, but quite hard only stick to itself and stringy and warps.	Can be used for printing, but quite hard only stick to itself and stringy and warps.	Good printing demonstrated but safety and health issues.	Often used as additive for better printing, not available in filament itself.	Good, high performance 3D printing material.	Been demonstrated, high temperatures though.	Printing demonstrated, again high temperatures.	Printing filament on the market	Printing filament on the market
**Interfacial properties w. carbon fibre**	Better interface properties for both mechanical performance and consolidation	IFSS of 19.3 MPa [34]	IFSS of 11-19 MPa [35]	Less than PA6, as PA6 improves adhesion [36]	Less than Nylon; carbon PETG filament has a lower strength than carbon Nylon filament [37]	Poor based on molecular structure	Strength reported of 1000 MPa (with nanodiamonds) [38]	IFSS of 20 MPa [39]	No studies found, expected low from molecular structure	No studies found, expected low from molecular structure	No studies found, expected low from molecular structure	Paper used coupling agent which was needed for interfacial adhesion [40]	IFSS of 100 MPa [41]	Similar to epoxy, IFSS around 80 MPa [42]	IFSS of around 40 MPa [43]	IFSS of around 50 MPa [44]	IFSS of around 50 MPa [44]
																	
					**Good property**			**Medium property**			**Poor property**						

**Table 2:** Trade-off study of polymer matrices for ADFRCs suitable for 3D printing.

Criteria	Weighting (%)	ABS	PETG	PLA	PBT	PCL	PSU	PESU	PA	LDPE	PP	PC	PEI	PVC	PPS	PEEK	HDPE
Processing temperature	12.5	3	2	3	3	3	1	1	2	3	3	1	1	3	1	1	2
Molding pressure	12.5	1	2	2	3	3	1	1	2	3	3	1	1	1	1	1	1
Costs	10.0	3	3	3	3	2	2	2	3	3	3	3	2	3	2	1	3
Glass transition temperature	7.5	2	2	2	2	1	3	3	2	1	1	3	3	2	2	3	1
Coefficient thermal expansion (CTE)	7.5	2	2	2	2	1	3	3	2	1	3	2	2	2	3	3	2
Thermal conductivity	7.5	2	2	1	2	1	3	3	2	3	1	2	2	2	2	2	3
Shrinkage	7.5	3	3	3	1	3	3	3	2	2	2	3	3	3	2	2	2
Printing capability	7.5	3	3	3	2	3	3	3	2	2	2	2	3	2	2	2	2
Interfacial properties w. carbon fibre	7.5	2	2	2	2	3	3	3	3	1	1	3	3	1	3	3	1
Specific heat capacity	7.5	2	2	1	2	2	2	2	2	3	2	1	2	1	2	2	3
Density	5.0	3	2	3	2	3	2	2	3	1	1	3	2	2	2	2	1
Crystallinity	5.0	3	3	2	2	2	3	3	2	2	2	3	3	2	2	2	1
Strength/stiffness	2.5	3	3	3	3	1	3	3	2	1	2	3	3	3	3	3	2
**TOTAL SCORE (/100):**		**79.2**	**77.5**	**76.7**	**76.7**	**75.8**	**75.8**	**75.8**	**74.2**	**73.3**	**71.7**	**70.8**	**70.8**	**68.3**	**64.2**	**63.3**	**62.5**
																
			**Good property**				**Medium property**				**Poor property**						

**Table 3:** Toho Tenax C124 carbon fibre properties.

Cut Length	Density	Filament Diameter	Tensile Strength	Tensile Modulus
3 mm	1.82 g/cm^3^	7 μm	4344 MPa	225 GPa

**Table 4:** Processing conditions for each ADFRC with a different thermoplastic matrix.

Material	T_low_	T_high_
PLA	170 °C	210 °C
Nylon	200 °C	260 °C
ABS	177 °C	260 °C
PETG	249 °C	288 °C

**Table 5:** Tensile test results of showing modulus, stress and strain at break and coefficient of variance (CoV).

Material	Temperature	Modulus (GPa)	CoV (%)	Stress (MPa)	CoV (%)	Failure Strain (%)	CoV (%)
**PLA**	Low	24.15	16.71	274.68	11.39	1.12	9.76
	High	28.00	13.69	351.54	22.64	1.20	15.45
**PA**	Low	15.71	10.67	215.83	8.45	1.41	3.85
	High	14.95	8.23	166.79	10.98	1.08	1.58
**ABS**	Low	11.24	8.63	76.76	17.08	0.70	11.93
	High	21.04	10.23	294.73	13.40	1.32	15.84
**PETG**	Low	27.12	3.05	300.49	14.61	1.09	12.54
	High	25.94	6.30	309.36	7.76	1.12	11.05

## References

[1] Mouritz A. (2012). Introduction to Aerospace Materials.

[2] Calignano F., Manfredi D., Ambrosio E.P., Biamino S., Lombardi M., Atzeni E., Salmi A., Minetola P., Iuliano L., Fino P. (2017). Overview on additive manufacturing technologies. Proc. IEEE.

[3] Vasiliev V.V., Razin A.F. (2006). Anisogrid composite lattice structures for spacecraft and aircraft applications. Compos. Struct..

[4] Stansbury J.W., Idacavage M.J. (2016). 3D printing with polymers: Challenges among expanding options and opportunities. Dent. Mater..

[5] Ning F., Cong W., Qiu J., Wei J., Wang S. (2015). Additive manufacturing of carbon fiber reinforced thermoplastic composites using fused deposition modeling. Compos. Part B Eng..

[6] Tekinalp H.L., Kunc V., Velez-Garcia G.M., Duty C.E., Love L.J., Naskar A.K., Blue C.A., Ozcan S. (2014). Highly oriented carbon fiber-polymer composites via additive manufacturing. Compos. Sci. Technol..

[7] Yang C., Tian X., Liu T., Cao Y., Li D. (2017). 3D printing for continuous fiber reinforced thermoplastic composites: Mechanism and performance. Rapid Prototyp. J..

[8] Blok L.G., Longana M.L., Yu H., Woods B.K.S. (2018). An investigation into 3D printing of fibre reinforced thermoplastic composites. Addit. Manuf..

[9] Brenken B., Barocio E., Favaloro A., Kunc V., Pipes R.B. (2018). Fused filament fabrication of fiber-reinforced polymers: A review. Addit. Manuf..

[10] Wang X., Jiang M., Zhou Z., Gou J., Hui D. (2017). 3D printing of polymer matrix composites: A review and prospective. Compos. Part B Eng..

[11] Justo J., Távara L., García-Guzmán L., París F. (2018). Characterization of 3D printed long fibre reinforced composites. Compos. Struct..

[12] Lacroix T., Tilmans B., Keunings R., Desaeger M., Verpoest I. (1992). Modelling of critical fibre length and interfacial debonding in the fragmentation testing of polymer composites. Compos. Sci. Technol..

[13] Mallick P.K. (1993). Fiber-Reinforced Composites: Materials, Manufacturing, and Design.

[14] Piggott M.R., Ko M., Chuang H.Y. (1993). Aligned short-fibre reinforced thermosets: Experiments and analysis lend little support for established theory. Compos. Sci. Technol..

[15] Yu H., Potter K.D., Wisnom M.R. (2014). A novel manufacturing method for aligned discontinuous fibre composites (High Performance-Discontinuous Fibre method). Compos. Part A Appl. Sci. Manuf..

[16] Tapper R.J., Longana M.L., Norton A., Potter K.D., Hamerton I. (2020). An evaluation of life cycle assessment and its application to the closed-loop recycling of carbon fibre reinforced polymers. Compos. Part B Eng..

[17] Tapper R.J., Longana M.L., Yu H., Hamerton I., Potter K.D. (2018). Development of a closed-loop recycling process for discontinuous carbon fibre polypropylene composites. Compos. Part B.

[18] Such M., Ward C., Potter K. (2014). Aligned Discontinuous Fibre Composites: A Short History. J. Multifunct. Compos..

[19] Yang D., Wu K., Wan L., Sheng Y. (2017). A Particle Element Approach for Modelling the 3D Printing Process of Fibre Reinforced Polymer Composites. J. Manuf. Mater. Process..

[20] Compton B.G., Post B.K., Duty C.E., Love L., Kunc V. (2017). Thermal analysis of additive manufacturing of large-scale thermoplastic polymer composites. Addit. Manuf..

[21] Bellehumeur C., Li L., Sun Q., Gu P. (2004). Modeling of Bond Formation Between Polymer Filaments in the Fused Deposition Modeling Process. J. Manuf. Process..

[22] Pollard D., Ward C., Herrmann G., Etches J. (2017). Filament Temperature Dynamics in Fused Deposition Modelling and Outlook for Control. Procedia Manuf..

[23] Turner B.N., Strong R., Gold S.A. (2014). A review of melt extrusion additive manufacturing processes: I. Process design and modeling. Rapid Prototyp. J..

[24] Sun Q., Bellehumeur C., Gu P. (2002). Effect of processing conditions on the bonding quality of FDM polymer filament. Solid Free. Fabr. Proc..

[25] McIlroy C., Olmsted P.D. (2017). Disentanglement effects on welding behaviour of polymer melts during the fused-filament-fabrication method for additive manufacturing. Polymer.

[26] de Gennes P.G. (1983). Entangled polymers. Phys. Today.

[27] Wool R.P., Yuan B.-L., McGarel O.K. (1989). Welding of polymer interfaces. Polym. Eng. Sci..

[28] Pabst W., Gregorová E., Berthold C. (2006). Particle shape and suspension rheology of short-fiber systems. J. Eur. Ceram. Soc..

[29] Mulholland T., Goris S., Boxleitner J., Osswald T., Rudolph N. (2018). Process-Induced Fiber Orientation in Fused Filament Fabrication. J. Compos. Sci..

[30] Choy C.L., Leung W.P., Kowk K.W., Lau F.P. (1992). Elastic moduli and thermal conductivity of injection-molded short-fiber–reinforced thermoplastics. Polym. Compos..

[31] Gibson I., Rosen D., Stuckers B. (2015). Additive Manufacturing Technologies: 3D Printing, Rapid Prototyping, and Direct Digital Manufacturing.

[32] Benedetti L., Brulé B., Decreamer N., Evans K.E., Ghita O. (2019). Shrinkage behaviour of semi-crystalline polymers in laser sintering: PEKK and PA12. Mater. Des..

[33] Granta Design Limited (2019). CES EduPack Software.

[34] Shipton P.D. (1988). The Compounding of Short Fibre Reinforced Thermoplastic Composites. Ph.D. Thesis.

[35] Wan Y.Z., Wang Y.L., Xu X.H., Li Q.Y. (2001). In Vitro Degradation Behavior of Carbon Fiber-Reinforced PLA Composites and Influence of Interfacial Adhesion. J. Appl. Polym. Sci..

[36] Li J., Zhang Y.F. (2010). The Tensile Properties of Short Carbon Fiber Reinforced ABS and ABS/PA6 Composites. J. Reinf. Plast. Compos..

[37] C3DXTech (2018). CarbonX Carbon Fiber Reinforced Filament. https://www.3dxtech.com/brands/CarbonXTM.html.

[38] Kausar A. (2015). Design and Study of Epoxy Composites based on Polycaprolactone and Nanodiamond Functionalized Carbon Fibers. Am. J. Polym. Sci. Eng..

[39] Dányádi L., Gulyás J., Pukánszky B. (2003). Coupling of carbon fibers to polycarbonate: Surface chemistry and adhesion. Compos. Interfaces.

[40] Chen Y., Wang X., Wu D. (2013). Recycled carbon fiber reinforced poly(butylene terephthalate) thermoplastic composites: Fabrication, crystallization behaviors and performance evaluation. Polym. Adv. Technol..

[41] Tang G., Zang Z., Chang D., Wei G., Wang D., Mi W., Yan W., Huang W. (2012). Study on the Interfacial Behavior of Clay-Coated Carbon Fiber-Reinforced PEI Composites. Polym. Plast. Technol. Eng..

[42] Gao S.L., Kim J.K. (2000). Cooling rate influences in carbon fibre/PEEK composites. Part 1. Crystallinity and interface adhesion. Compos. Part A Appl. Sci. Manuf..

[43] Liu B., Liu Z., Wang X., Zhang G., Long S., Yang J. (2013). Interfacial shear strength of carbon fiber reinforced polyphenylene sulfide measured by the microbond test. Polym. Test..

[44] Hartwig G., Jäger H., Knaak S., Hartwig G., Evans D. (1986). Interlaminar Shear Strength of Carbon-Fibre Reinforced Thermoplastics Polycarbonate and Polysulfone. Nonmetallic Materials and Composites at Low Temperatures.

[45] Weng Z., Wang J., Senthil T., Wu L. (2016). Mechanical and thermal properties of ABS/montmorillonite nanocomposites for fused deposition modeling 3D printing. Mater. Des..

[46] Biron M. (2007). Thermoplastics and Thermoplastic Composites.

[47] Yumitori S., Arao Y., Tanaka T., Naito K., Tanaka K., Katayama T. (2013). Increasing the interfacial strength in carbon fiber/polypropylene composites by growing CNTs on the fibers. WIT Trans. Model. Simul..

[48] Chong S., Pan G.T., Khalid M., Yang T.C.K., Hung S.T., Huang C.M. (2017). Physical Characterization and Pre-assessment of Recycled High-Density Polyethylene as 3D Printing Material. J. Polym. Environ..

[49] Cicala G., Ognibene G., Portuesi S., Blanco I., Rapisarda M., Pergolizzi E., Recca G. (2018). Comparison of Ultem 9085 used in fused deposition modelling (FDM) with polytherimide blends. Materials.

[50] Zaldivar R.J., Witkin D.B., McLouth T., Patel D.N., Schmitt K., Nokes J.P. (2017). Influence of processing and orientation print effects on the mechanical and thermal behavior of 3D-Printed ULTEM^®^9085 Material. Addit. Manuf..

[51] Telford R., Peeters D., Oliveri V., Zucco G., Jones D., O’Higgins R., Weaver P.M. Enhanced Buckling Performance of a Stiffened, Variable Angle Tow Thermoplastic Composite Panel. Proceedings of the 2018 AIAA/ASCE/AHS/ASC Structures, Structural Dynamics, and Materials Conference.

[52] Comer A.J., Ray D., Obande W.O., Jones D., Lyons J., Rosca I., O’Higgins R.M., McCarthy M.A. (2015). Mechanical characterisation of carbon fibre-PEEK manufactured by laser-assisted automated-tape-placement and autoclave. Compos. Part A Appl. Sci. Manuf..

[53] Ray D., Comer A.J., Lyons J., Obande W., Jones D., O’Higgins R.M., McCarthy M.A. (2015). Fracture toughness of carbon fiber/polyether ether ketone composites manufactured by autoclave and laser-assisted automated tape placement. J. Appl. Polym. Sci..

[54] Berretta S., Davies R., Shyng Y.T., Wang Y., Ghita O. (2017). Fused Deposition Modelling of high temperature polymers: Exploring CNT PEEK composites. Polym. Test..

[55] Chen B., Berretta S., Davies R., Ghita O. (2019). Characterisation of carbon fibre (Cf)—Poly Ether Ketone (PEK) composite powders for laser sintering. Polym. Test..

[56] Stoeffler K., Andjelic S., Legros N., Roberge J., Schougaard S.B. (2013). Polyphenylene sulfide (PPS) composites reinforced with recycled carbon fiber. Compos. Sci. Technol..

[57] Kishore V., Chen X., Ajinjeru C., Hassen A.A., Lindahl J.M., Failla J., Kunc V., Duty C.E. Additive manufacturing of high performance semicrystalline thermoplastics and their composites. Proceedings of the Solid Freeform Fabrication Symposium—An Additive Manufacturing Conference.

[58] Zhansitov A.A., Khashirova S.Y., Slonov A.L., Kurdanova Z.I., Shabaev A.S., Khashirov A.A., Mikitaev A.K. (2017). Development of technology of polysulfone production for 3D printing. High Perform. Polym..

[59] Markforged (2016). High Strength 3D Printing with Continuous Fibres. https://markforged.com/.

[60] 3D4Makers 3D4Makers Filament Engineers. https://www.3d4makers.com/.

[61] Valentin D., Paray F., Guetta B. (1987). The hygrothermal behaviour of glass fibre reinforced Pa66 composites: A study of the effect of water absorption on their mechanical properties. J. Mater. Sci..

[62] Cox H.L. (1952). The elasticity and strength of paper and other fibrous materials. Br. J. Appl. Phys..

[63] Longana M.L., Ong N., Yu H., Potter K.D. (2016). Multiple Closed Loop Recycling of Carbon Fibre Composites with the HiPerDiF (High Performance Discontinuous Fibre) Method. Compos. Struct..

[64] Teijin Carbon (2020). Tenax® Short Fiber Product Data Sheet Chopped Fiber with Thermoplastic Sizing. https://www.teijincarbon.com/products/tenaxr-carbon-fiber/tenaxr-short-fibers.

[65] Campbell F.C. (2004). Manufacturing Processes for Advanced Composites.

[66] Gebart B.R. (1992). Permeability of Unidirectional Reinforcements for RTM. J. Compos. Mater..

[67] Goh K. (2016). Discontinuous-Fibre Reinforced Composites.

[68] Wagner T. (2016). ParticleSizer. https://imagej.net/ParticleSizer.

[69] Technology Outlet (2019). Premium PET-G Carbon Fibre Filament. https://technologyoutlet.co.uk/products/technology-outlet-premium-pet-g-carbon-fibre-filament.

[70] Technology Outlet (2019). Premium PLA Carbon Fibre Filament. https://technologyoutlet.co.uk/products/carbon-pla-3d-printer-filament.

